# The 2025 Nobel Prize in Physiology or Medicine Honors the Immune Peacekeepers

**DOI:** 10.3389/ti.2025.15767

**Published:** 2025-11-24

**Authors:** Julien Zuber, Hannah Kaminski, the Scientific Committee of the Société Francophone de Transplantation

**Affiliations:** 1 Département des Maladies du Rein et du Métabolisme, Transplantation et Immunologie Clinique, Hôpital Necker, Assistance Publique-Hôpitaux de Paris, Paris, France; 2 Inserm UMR_S 1151, Institut Necker Enfants Malades, Université Paris Cité, Paris, France; 3 Département de Néphrologie, Transplantation, Dialyse et Aphérèse, Hôpital Pellegrin, Bordeaux, France; 4 Université de Bordeaux, CNRS, ImmunoConcEpT, UMR_5164, Inserm ERL U1303, Equipe Labellisée par la Ligue Nationale Contre le Cancer, Bordeaux, France

**Keywords:** 2025 Nobel Prize, regulatory T cells, Sakaguchi, Brunkow, Ramsdell, FOXP3

In an era marked by global conflict, polarization, and societal fragmentation, the Nobel Committee has chosen to honor three scientists (Figure 1) for their discovery of key cellular players involved in *Immune Tolerance* and homeostatic regulation. In their 1960 Nobel Lecture, Medawar and Burnet defined immune tolerance as “*a state of indifference or non-reactivity towards a substance that would normally be expected to excite an immunological response*”, a definition that remains largely unchanged today (Glossary).

**FIGURE 1:**
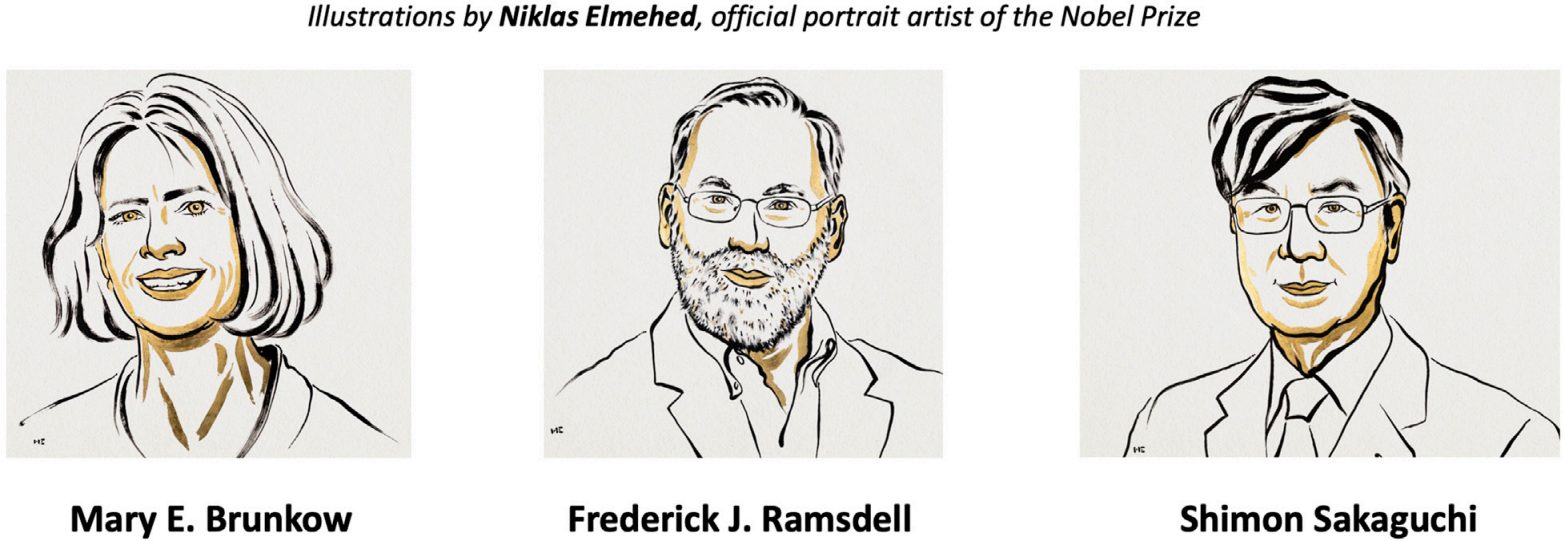
The Three Laureates of the 2025 Nobel Prize in Physiology or Medicine Dr. Brunkow, PhD, an American molecular biologist, currently holds the position of Senior Program Manager at the Institute for Systems Biology (ISB) in Seattle. Her Nobel-winning work was carried out at Celltech in Bothell, Washington. Dr. Ramsdell, PhD, an American immunologist, is the Chief Scientific Officer at Sonoma Biotherapeutics in San Francisco. His award-winning research also took place at Celltech in Bothell. Dr. Sakaguchi, MD, PhD, a Japanese immunologist, serves as a Distinguished Professor at Osaka University. His honored contributions were made at the Institute for Frontier Medical Sciences at Kyoto University.

The laureates’ seminal work led to the discovery and characterization of regulatory CD4^+^ FOXP3^+^ T cells (Tregs), now widely recognized as central orchestrators of peripheral immune tolerance, alongside other innate and adaptive immune cells. This breakthrough has laid the foundation for innovative therapeutic strategies across a broad range of clinical applications.

## The First “Giant” Steps Forward

Shimon Sakaguchi was the first to provide decisive and widely accepted insights into these cells in 1995, turning the page on the previously ill-defined and controversial “suppressive T cells” of the 1980s. His seminal publication identified the constitutive expression of the high-affinity interleukin-2 receptor as a major phenotypic marker of regulatory T cells Tregs [1]. He also demonstrated their capacity to prevent autoimmunity in a mouse model [1].

In 2001, Mary Brunkow and Fred Ramsdell established a critical link between the human IPEX syndrome (Immune dysregulation, Polyendocrinopathy, Enteropathy, X-linked) and the murine Scurfy phenotype, both marked by severe autoimmune manifestations. They identified a shared genetic origin: mutations in the *FOXP3* gene located on the X chromosome [2, 3]. The emergence of FOXP3 as a master regulator of immune tolerance immediately raised compelling questions about its role in Tregs.

In 2003, Shimon Sakaguchi, Fred Ramsdell, and Alexander Rudensky independently, and almost simultaneously, published landmark studies demonstrating the essential role of FOXP3 in defining the identity and function of regulatory T cells [4–6].

**FIGURE 2:**
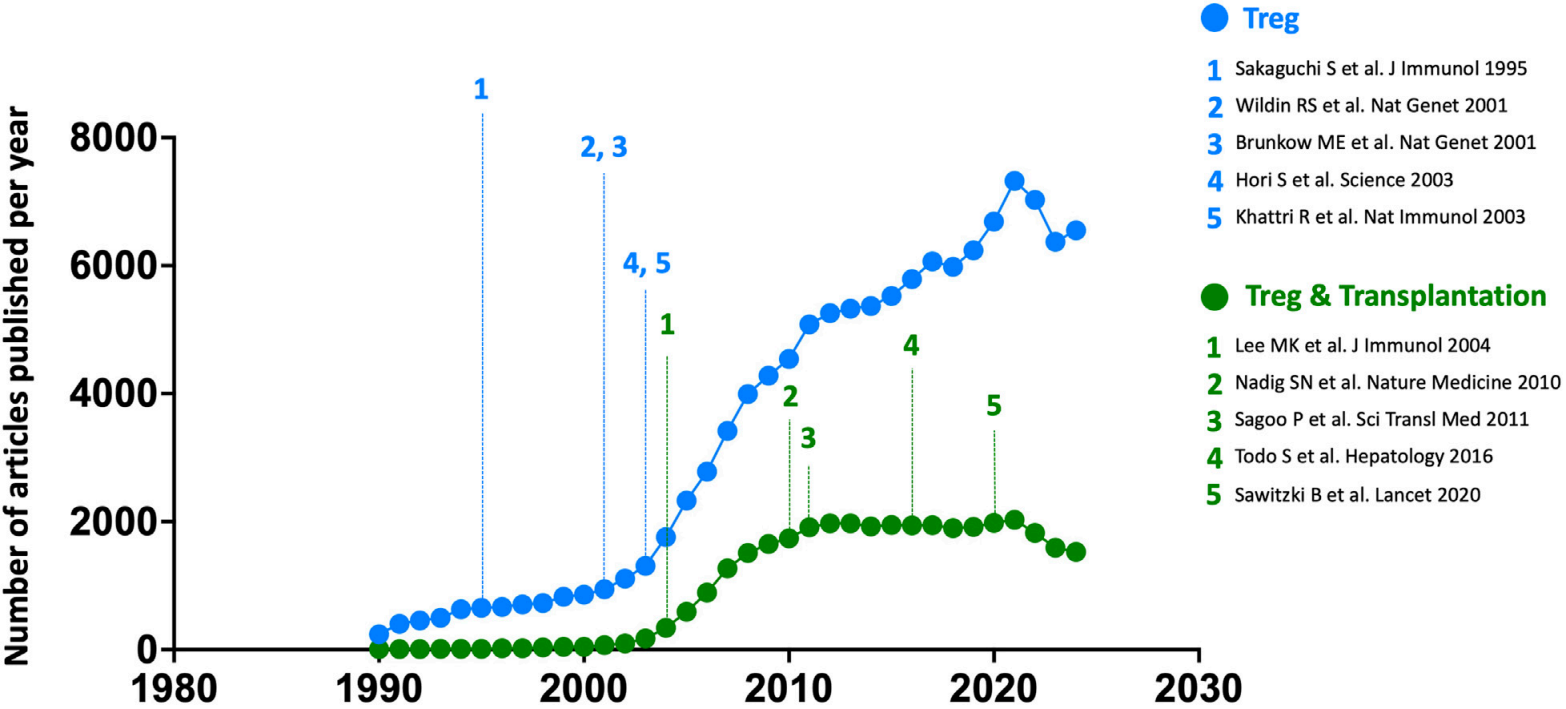
Annual number of all articles published on Tregs, across all fields (blue) and specifically focused on transplantation (green), from 1990 to 2024. The five seminal papers by the three Nobel Prize laureates are numbered in blue along the chronological timeline, while five landmark studies in the field of transplantation are highlighted in green. Bibliographic data were extracted from the Web of Science platform (Clarivate Analytics) using the keywords [FOXP3] or [REGULATORY T CELL] for all fields, and [FOXP3] or [REGULATORY T CELL] combined with [TRANSPLANTATION] for articles specifically focused on Tregs in transplantation.

This discovery marked the beginning of a remarkable surge of interest in these cells (Figure 2), a trend further accelerated by the development of novel molecular tools and the emergence of murine models enabling selective gene expression or deletion in Tregs. Tregs originate from two distinct developmental pathways, depending on the ontogenetic timing of their commitment to the regulatory lineage: either thymic-derived (tTregs) or peripherally induced (pTregs) [7–9]. The former possess a highly self-reactive T cell receptor repertoire and primarily function to maintain self-tolerance and prevent autoimmunity. In contrast, pTregs differentiate in response to exogenous antigens within peripheral tissues, particularly in environments enriched in TGF-β. Notably, pTregs are key regulators of immune responses at mucosal interfaces, where they suppress immune reactions to dietary antigens and commensal microbiota [7, 8]. They also play a crucial role in preventing maternal immune responses against paternal antigens expressed by the fetus [10].

In this context, the evolutionary conservation of a specific regulatory element within the *FOXP3* gene among eutherian (placental) mammals, but not in marsupials or oviparous mammals, underscores the essential role of pTregs in mammalian evolution, ensuring maternal tolerance necessary for successful gestation and complete fetal development [10].

## Tregs are Ubiquitous in Human Immunopathology

**FIGURE 3:**
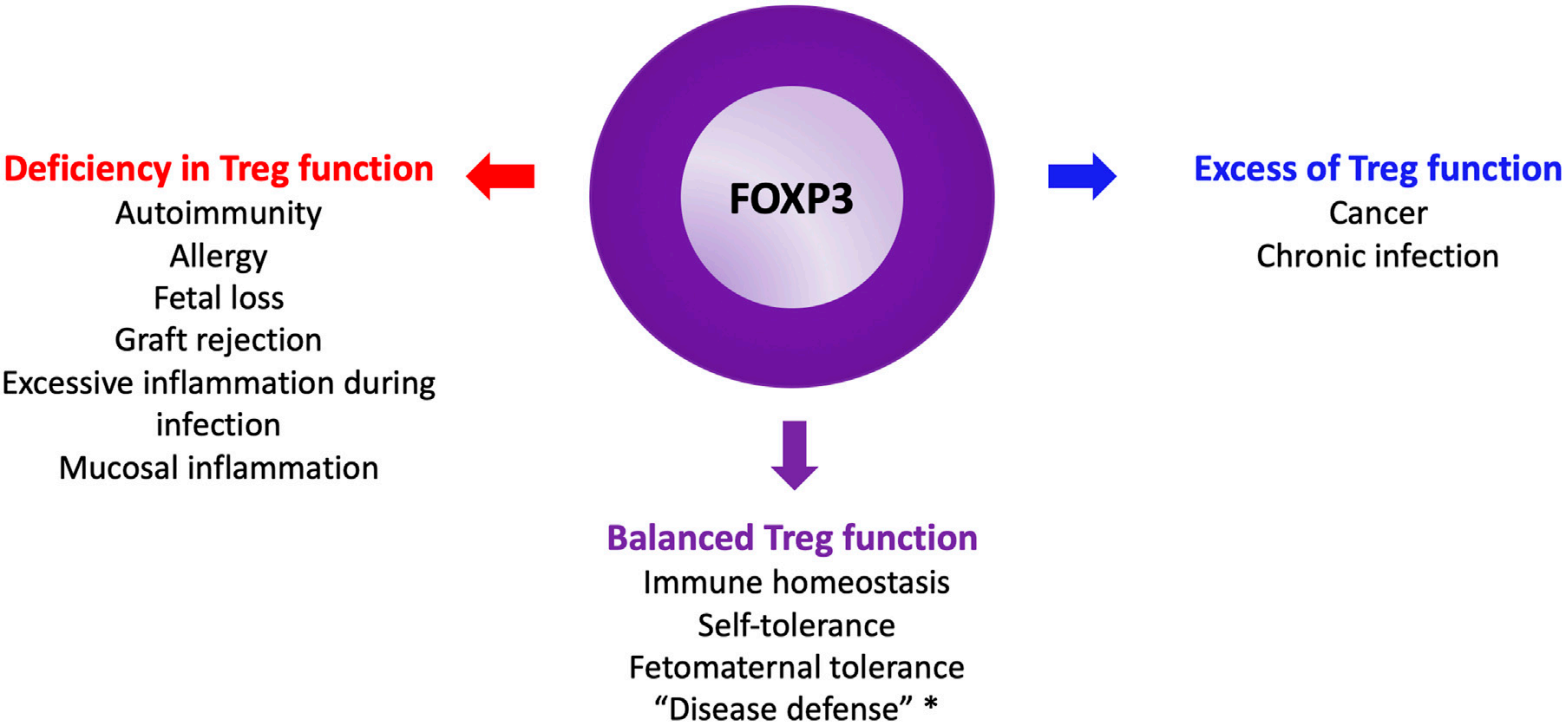
The Yin and the Yang of Tregs. *: see glossary for definition.

Human Treg subpopulations were first well characterized in a landmark paper by Makoto Miyara in Sakaguchi’s laboratory [11]. Over the past two decades, dysregulated human regulatory T cell (Treg) function, whether excessive or insufficient, has been implicated across the full spectrum of immunopathology (Figure 3).

Beyond the extreme case of IPEX syndrome, Treg deficiency has been identified in various autoimmune diseases [15]. Shimon Sakaguchi’s group demonstrated that single nucleotide polymorphisms linked to common autoimmune disorders are predominantly located in demethylated regions specific to naïve Tregs [16]. These regions shape the unique transcriptomic and epigenetic identity of Tregs, suggesting that impaired development or function of natural Tregs is a major driver of autoimmunity [16].

During healthy pregnancy, the Treg population expands alongside increased bioavailability of interleukin-2 (IL-2), a cytokine essential for Treg homeostasis [17]. A collapse in IL-2 signaling at the end of gestation coincides with the emergence of an inflammatory signature associated with parturition [17]. A recent study identified a subset of highly suppressive, activated CCR8-expressing Tregs at the decidual interface during the first trimester [18]. This population is reduced in recurrent pregnancy loss in humans and in murine models of spontaneous abortion. In mice, selective depletion of CCR8^+^ decidual Tregs precipitates fetal loss, while their adoptive transfer protects against spontaneous abortion [18].

In the context of organ transplantation, Tregs play a pivotal role in suppressing alloimmune responses [19]. Their involvement in maintaining and propagating transplant tolerance has been well demonstrated in experimental models, offering a cellular basis for the phenomenon of *Infectious Tolerance* (Glossary) [12, 13]. In humanized mouse models, human Tregs can suppress both acute and chronic rejection, with enhanced efficacy when enriched for donor antigen-specificity [20, 21]. In clinical transplantation, the expansion and/or graft infiltration of Tregs in patients who achieve operational tolerance, either spontaneously or through therapeutic intervention [22], highlights their potential to reduce the need for long-term immunosuppression.

One of the earliest insights into the role of Tregs in anti-infectious immunity came from Shohei Hori, a key contributor to Sakaguchi’s seminal 2003 study [4]. Hori demonstrated that Tregs play a crucial role in modulating the clinical manifestation of pneumocystis pneumonia by limiting inflammation [23]. In their absence, the infection took on a highly inflammatory and lethal course. Similarly, Rudensky’s group identified amphiregulin-expressing Tregs involved in tissue repair; their impairment led to severe lung damage during influenza infection [24]. These findings support the concept of *Disease Tolerance* (see Glossary), where the host aims to both control the pathogen and minimize immune-mediated tissue damage [14]. Conversely, in chronic infections Tregs can be detrimental by impairing pathogen clearance [25].

Finally, a population of highly suppressive, activated CCR8^+^ Tregs, similar to those found in the decidua, accumulate at tumor sites and contribute to the creation of an immune-privileged environment that enables cancer immune evasion [26]. Shimon Sakaguchi has shown that targeted depletion of CCR4+ Tregs or CCR8^+^ Tregs can restore a robust, memory-driven anti-tumor immune response [27, 28].

## Toward Targeted Therapies

The field of oncology has embraced targeted therapies against intratumoral Tregs. The 2018 Nobel Prize in Physiology or Medicine was awarded to James Allison and Tasuku Honjo for their discoveries of the immune checkpoints CTLA-4 and PD-1, which laid the foundation for revolutionary cancer immunotherapies. While PD-1 inhibitors primarily target intratumoral CD8^+^ T cells, CTLA-4 blockade mainly disrupts Treg suppressive mechanisms [29]. In this regard, anti-CTLA-4 antibodies represent the first Treg-targeted immunotherapies. Another strategy involves depleting Tregs using anti-CCR4 antibodies, such as mogamulizumab, currently used to treat cutaneous lymphomas. Even more promising are anti-CCR8 therapies, with the potential to transform cancer immunotherapy [30].

Conversely, several academic and industrial research groups are developing novel therapeutic strategies to induce stable, suppressive Tregs from conventional T cells. Until recently, culturing T cells with TGF-β and IL-2 yielded only transient FOXP3 expression, resulting in an unstable regulatory phenotype. In this context, Shimon Sakaguchi’s laboratory recently demonstrated the conversion of antigen-specific conventional T cells into stable, suppressive Tregs both *in vitro* and *in vivo* (in mice), either by inhibiting cyclin-dependent kinases 8 and 19 or abrogating CD28 signaling [31, 32]. Other teams are exploring chromatin-modifying agents to establish the epigenetic landscape characteristic of *bona fide* Tregs, essential for maintaining regulatory identity [33]. The therapeutic potential of this emerging class of immunomodulators is highly promising.

IL-2-based therapies exploit the high-affinity IL-2 receptor expression characteristic of Tregs, resulting in heightened sensitivity to low-dose IL-2 [34]. While low-dose IL-2 has shown clinical benefit in treating chronic graft-versus-host disease [35], it has also led to graft rejection in kidney (NCT02417870) and liver transplant [36] recipients due to limited specificity for Tregs. This narrow therapeutic window has spurred interest in IL-2 muteins: genetically engineered IL-2 variants designed to selectively activate Tregs [34, 37]. These modified cytokines are being developed primarily for autoimmune diseases, though they also hold promise for solid organ transplantation [34].

Regulatory cell therapy is attracting growing interest in treating autoimmune diseases, hematopoietic stem cell transplantation (HSCT), and solid organ transplantation. The Orca-T cell product, which includes donor-derived Tregs, has achieved breakthrough results in phase 2 [38] and subsequent phase 3 (NCT05316701) clinical trials, demonstrating a significantly lower incidence of moderate-to-severe chronic GVHD at 1 year among patients undergoing allogeneic HSCT. Orca-T is poised to become the first FDA-approved Treg-based cell therapy. In kidney transplantation, results from the ONE Study demonstrated the feasibility and safety of an autologous, polyclonal Treg therapy in kidney transplant recipients [39]. The findings suggest potential benefits, including reduced immunosuppressive requirements and a lower incidence of opportunistic infections [39]. In liver transplantation, a Japanese study showed that immunosuppressive drugs could be successfully discontinued following post-transplant cyclophosphamide pulses and donor-specific Treg therapy, with sustained results over long-term follow-up [40, 41].

Genetic enhancement of Tregs represents a promising strategy to potentiate regulatory cell therapy [42]. For example, Tregs can be redirected to the graft by engineering them to express a chimeric antigen receptor (CAR) specific for a donor-derived antigen, such as HLA-A2 [43]. Two clinical trials investigating HLA-A2–specific CAR-Tregs are currently underway in kidney (STEADFAST, NCT04817774) and liver (LIBERATE, NCT05234190) transplantation. Additionally, Tregs can be rendered resistant to tacrolimus through targeted deletion of the FKBP12 gene, preserving their function and proliferation in patients under immunosuppressive therapy [44]. Lastly, transgenic expression of an IL-2 mutein can enhance Treg expansion and suppressive capacity [45].

In summary, 30 years after the foundational work that shaped our modern understanding of regulatory T cells, their medical implications have proven profound, especially in organ transplantation. We extend our warmest thanks to the three laureates for their groundbreaking contributions and congratulate them on this well-deserved recognition.

## Data Availability

The original contributions presented in the study are included in the article/supplementary material, further inquiries can be directed to the corresponding author.

## Appendix

Please see the glossary for definition.

Glossary:

1- Immune tolerance refers to the immune system’s ability to remain unresponsive to molecules, cells, or tissues that would otherwise trigger a response. It involves various mechanisms that help distinguish between self and non-self, while also preventing excessive or inappropriate reactions to environmental factors such as dietary antigens and gut microbiota. Depending on where it is induced, immune tolerance is classified as either central tolerance, which occurs in the thymus and bone marrow, or peripheral tolerance, which takes place primarily in lymph nodes and other tissues.
2- Infectious tolerance refers to the capacity of *bona fide* Tregs to convert effector cells into new Tregs, thereby extending immune tolerance from one antigen to another. This mechanism supports the ongoing induction of tolerance in new cohorts of T cells over the lifespan of a tolerated graft. The concept was first introduced by Hermann Waldmann in 1993 [12], and was later linked to regulatory T cells in 2011 [13].
3- Disease tolerance refers to a paradigm proposed by Ruslan Medzhitov, in which an important defense strategy against infection involves mitigating bystander tissue injury caused by pathogen-specific immune responses [14].
